# Fractal dimension of particle-size distribution and their relationships with alkalinity properties of soils in the western Songnen Plain, China

**DOI:** 10.1038/s41598-020-77676-w

**Published:** 2020-11-26

**Authors:** Yufeng Bai, Yan Qin, Xinrui Lu, Jitao Zhang, Guoshuang Chen, Xiujun Li

**Affiliations:** 1 Northeast Institute of Geography and Agroecology, Chinese Academy of Sciences, Changchun, 130102 People’s Republic of China; 2 University of Chinese Academy of Sciences, Beijing, 100049 People’s Republic of China

**Keywords:** Solid Earth sciences, Petrology, Environmental sciences, Environmental impact

## Abstract

The purpose of this study was to identify the fractal dimension and their relationships with alkalinity properties of soils, and to evaluate the potential of fractal dimension as an indicator of alkalinity properties of soil. Six soils with an increasing salinity (electrical conductivity was 0.09, 0.18, 0.62, 0.78, 1.57 and 1.99 dS m^−1^, respectively) were selected from the western part of the Songnen Plain (China). Salt content, exchangeable sodium percentage, sodium adsorption ratio and other properties of the soils were determined and the soil particle-size distribution (0–2000 μm) was measured using a laser diffraction particle size analyser. Our results show that the overall fractal dimension of the selected soils ranged from 2.35 to 2.60. A linear regression analysis showed a significant negative correlation between fractal dimension and the amount of coarse sand and fine sand (r =  − 0.5452, *P* < 0.05 and r =  − 0.8641, *P* < 0.01, respectively), and a significant positive correlation with silt and clay (r = 0.9726, *P* < 0.01 and r = 0.9526, *P* < 0.01, respectively). Thus, soils with higher silt and clay content have higher fractal dimension values. Strong linear relationships between fractal dimension and salt content (*P* < 0.05), in particular a very significant positive relationship with HCO_3_^−^ (*P* < 0.01), also exist. It is therefore possible to conclude that a soil’s fractal dimension could serve as a potential indicator of soil alkalization and the variability in alkaline soil texture.

## Introduction

Fractal geometry theory has been used to describe natural bodies and phenomena displaying complicated shapes and self-similar characteristics^1,2^. Soil is a porous medium with a varied particle-size distribution as well as having irregular shapes and a self-similar structure, hence displaying fractal characteristics^3,4^. At present, several researchers have explored different ways of analysing the fractal dimension of materials. Early work by Turcotte^2^ found that fractal concepts can be used as a measure of the fragility of fragmented material^2^. Tyler and Wheatcraft^4^ and Yang et al.^5^ developed a mass-based distribution model to calculate the fractal dimension of particle-size distribution. Following this, researchers have studied features of the fractal dimension of different soils and found that fractal theory was a useful tool for describing the soil particle-size distribution (PSD) and structure^6,7^. However, with more comprehensive studies, the hypothesis of the same density of soil particles with different sizes has been questioned^8,9^. Based on previous research, Wang et al.^9^ developed a volume-based model to quantify the fractal dimension of PSD. The volume-based model has been widely used in particle size analysis, as the parameters and data required can be easily and accurately obtained using a laser particle-size analyser, thus allowing the assumption of the same density of soil particles of different sizes to be avoided^10^. Yang et al.^11^ compared the volume-based and mass-based models, and found the former underestimated the clay content. To date, fractal theory has been used to describe soil particle-size distribution, aggregate-size distribution, and pore-size distribution^12^. Compared with traditional methods for characterizing soil particle-size distribution, fractal theory describes it well because it can characterize the local shape, size, structure and function of the material and any similarities between the part and the whole^10^.

Fractal theory has been developed and widely used as a “bridge” for describing and quantifying the correlation between the distribution of soil particle-size and the physical and chemical attributes of soils. Several studies have shown that the fractal dimension of the soil PSD was directly proportional to the silt and clay content, and inversely proportional to the sand content^7^. Differences in soil particle-size distribution affects the soil’s bulk density^10,13^, water movement^14–16^, and soil nutrients^17,18^. Thus, the fractal dimension of a soil has a close relationship with these parameters. Su et al.^19^ studied the plough layer of croplands and found the contents of organic carbon and total nitrogen had a positive correlation with the soil’s fractal dimension. Ersanin et al.^20^ compared twenty-two soils with different textures and parent materials, where the results indicated that the relationship between fractal dimension and a soil’s specific surface area and cation exchange capacity can be well described by a second-degree polynomial regression model. Given fractal dimension is sensitive to property changes under natural conditions and anthropogenic activities, fractal dimension has been used as effective indicators of desertification^21,22^, plant invasion^18^, and vegetation restoration^23^.

Alkaline soils represent one of the three salt-affected soil types (saline, sodic or alkaline soil and saline–alkaline soil), which have an electrical conductivity found from a derived saturated paste extract (ECe) < 4 dS m^−1^ and sodium adsorption ratio (SAR) ≥ 13^24^. High pH and excessive exchangeable sodium are the most obvious attributes of alkaline soils^25^. Under natural condition, the force that holds clay particles together is greatly decreased when clay particles with negative charges absorb excessive sodium ions^26,27^. Then, when the soil gets dry, these dry particles are dispersed into individual clay particles and form dense layers which then block pores^28,29^. This process weakens the aggregation of soil and causes structural collapse, leading to limited water and air flow through the soil. As demonstrated by Ding et al.^30^, the swelling strength in a Mg^2+^ solution is much weaker than that in a Na^+^ solution, and inversely much greater infiltrability values arise in Mg^2+^ solutions than in Na^+^ solutions. Apparently, sodium ions play a critical role in the swelling and dispersing of clay particles into smaller individual particles. While it has been found that there is a significant correlation between the fractal dimension and salt content and salt composition of soils^31,32^, there has been little attention paid to alkaline soils, especially soils containing large amounts of sodium bicarbonate. It is therefore unclear whether there is a relationship between the dispersive sodium and fractal dimension.

The Songnen Plain is one of the world’s major saline–alkaline land areas^33^. Soil salinization has become a critical factor, restricting the development of the regional economy and posing a major challenge to environmental protection^34^. Determining the correlation between fractal dimension and alkalinity properties of soils would therefore be helpful in planning the optimal use of regional water and soil resources. Therefore, the objectives of this study were to: 1) determine the fractal dimension of soil with different degrees of alkalinity, 2) quantify the relationship between soil fractal dimension and soil properties, and 3) assess the potential of using fractal dimension as an indicator the properties of alkaline soil.

## Materials and methods

### Study area

**Figure 1:**
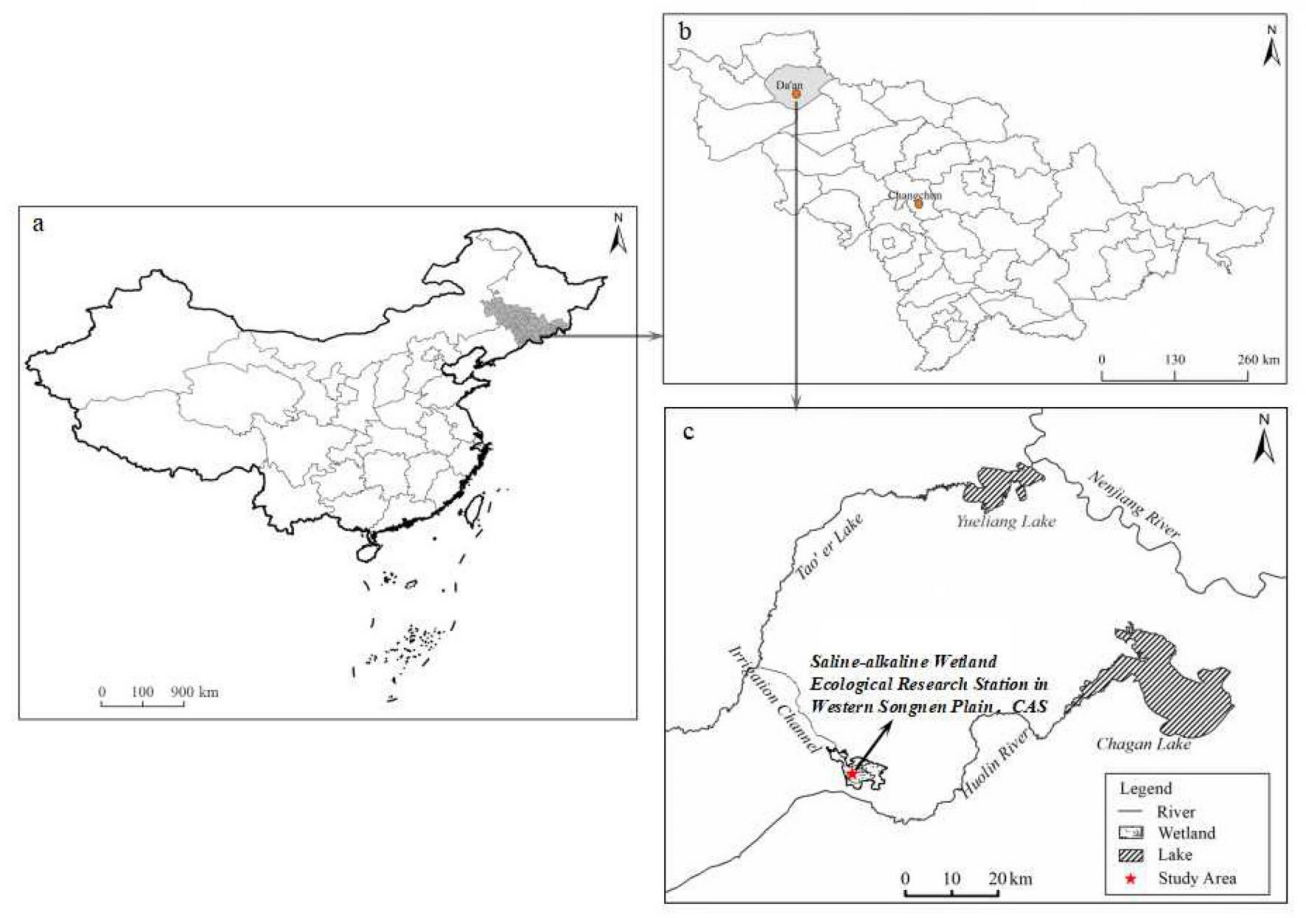
Map of region and study area. (**a**) Location map of China. (**b**) Location map of Songnen Plain, China. (**c**) Location map of the study area in Western Songnen Plain in Northeast China. This map was generated by ArcGIS 10.2 (http://www.arcgis.com/features/index.html).

The study area is located in the Saline–alkaline Wetland Ecological Experiment Station in the Western Songnen Plain in Northeast China (123° 08′ to 123° 21′ E and 44° 57′ to 45° 45′ N, Fig. 1). This region has an elevation of 110 m to 140 m a.s.l, where the terrain is a low flood plain, with slope gradients ranging from 1/7000 to 1/10,000. It is located within a transition zone between semi-arid to semi-humid and has a continental monsoon climate. Annual rainfall is approximately 396 mm with 73% of it occurring between June and August. The average annual evaporation is 1817 mm, and the average temperature is 2–5 °C^35^. The study area is one of the three major salt-affected areas in the world, with the area of salt-affected soil being close to 3.42 × 10^6^ hectares, representing approximately 19.7% of the plain’s total area. The salt composition is dominated by NaHCO_3_ and Na_2_CO_3_, and it is a typical alkaline soil^35^. Salt-affected soils in the study area are divided into three categories: saline, sodic (alkaline) and saline-sodic meadow soils. Saline soil is mainly distributed around the low flood plain, which has a shallow groundwater level was 1.0–1.5 m. This saline soil shows a patchy distribution, and has an excessive soluble salt content ranging from 1 to 2% at the surface layer. Alkaline soil is distributed about the relatively high levels of the micro-topography, where the groundwater level is 1.5–2.0 m. The salt composition is dominated by sodium bicarbonate, with a pH of more than 9.0 and highly dispersed soil colloids. Saline-sodic meadow soil formed under the influence of micro-topography and is distributed in patches amongst saline and alkaline soils, with the groundwater level being more than 2.0 m below the surface. The zonal vegetation is dominated by meadow plants, with the dominant species being *Aneurolepidium Chinese* and *Chloris virgata*. Azonal vegetation is mainly dominated by salt-tolerant plants such as *Phragmites australics*, *Suaeda glauca* and *Puccinellia tenuiflora*. The azonal vegetation presents a mosaic distribution amongst the zonal vegetation^34^.

### Sampling design and analysis

The distribution of plant communities is a function of the area’s climate and soil, therefore, different plant communities reflect the differences in their environment^36,37^. In the case of this work, halophytes can reflect the degree of salinization and salt composition of an area^38,39^. Several researchers have attempted to explain the vegetation distribution of the Songnen Plain^40,41^. The distribution of plant communities in saline–alkaline land is greatly affected by micro-topography, and different topographies often see the distinctive distribution of plant communities^34^. For example, *Suaeda glauca* and *Artemisia suaeda* communities are distributed around depressions, where generally, *Artemisia suaeda* is found outside of the depression while *Suaeda glauca* is distributed within it. In the local depression or slightly watery alkaline spots, *Pucinellia tennislora* communities dominant. Halophytes, such as *Suaeda glauca* and *Artemisia suaeda,* grow on the alkaline spots near lakes, while *Aneurolepidium Chinese* and weeds are mainly distributed across the flat terrain. *Phragmites australis* communities gradually increase in extent as the soil moisture increase, and sometimes the vegetation composition will transform from grassland formed by *Aneurolepidium Chinese* to marsh formed by *Phragmites australis*. In areas where alkaline spots are exposed to the surface, there is often a barren portion where the grass is bare. Some mounds that are far away from river channels, and are low in water content may be reclaimed as arable land and are mainly used to cultivate corn in this region.

Six sampling sites (i.e., cropland (CL, disturbed by tillage and crops), *Aneurolepidium Chinese* (AC), *Phragmites australis* (PA), *Chloris virgata* (CH), *Suaeda glauca* (SG), and bare land made up of saline-sodic soil (BL)) were selected at study area in August 2019. As shown in Table 1, the order of the six sampling sites exhibited an increasing trend in salinization. Except for CL, all sites were naturally salt-affected soils without disturbance by tillage or grazing. Surface plant residue and stones were carefully removed, and three soil samples were collected using a stainless-steel auger to a depth of 30 cm at each sampling site. All collected soils were placed in polyethylene bags and labelled. After air-drying at room temperature for approximately 20 days, the samples were sieved through a 2-mm sieve to remove coarse debris, roots and stones. A portion of each soil sample was used for determining the particle-size distribution (PSD) by a laser diffraction particle-size analyser (Malvern Instruments 2000, Malvern, England). The range of particle sizes that can be measured by this instrument is 0.02–2000 μm, which provides a continuous volume percentage of particle sizes, with a repeat error of < 2%. Following the United States Department of Agriculture Soil Taxonomy, the particle sizes were classified into three grades, clay (< 2 μm), silt (2–50 μm) and sand (50–2000 μm). Then, in order to yield an appropriate number of data points per sample to allow the linear fitting of the fractal dimension in the next steps, the three grades are artificially subdivided into twenty-four sub-intervals making use of the Mastersizer 2000 software (Ver. 5.61, Malvern Instrument Ltd., England). These subdivisions are: 0–0.1, 0.1–0.5, 0.5–1.0, 1–2, 2–3, 3–4, 4–5, 5–6, 6–7, 7–10, 10–20, 20–30, 30–50, 50–80, 80–100, 100–150, 150–200, 200–250, 250–300, 300–500, 500–800, 800–1000, 1000–1500, and 1500–2000 μm.

**Table 1:** Physical and chemical properties of soil samples in different plant communities.

Soil properties	Different communities						Statistical parameter	
	CL	AC	PA	CH	SG	BL	*SD*	*CV/%*
pH	8.68 ± 0.06	9.31 ± 0.19	9.90 ± 0.02	10.35 ± 0.17	10.23 ± 0.05	10.31 ± 0.02	0.67	6.85
EC_e _(dS/m)	0.09 ± 0.00	0.18 ± 0.03	0.62 ± 0.01	0.78 ± 0.01	1.99 ± 0.10	1.57 ± 0.04	0.76	87.33
ENa^+^ (mg kg^−1^)	1.61 ± 0.09	4.83 ± 0.06	64.86 ± 5.17	83.03 ± 6.74	165.83 ± 9.94	150.88 ± 10.12	69.87	89.01
ESP (%)	1.09 ± 0.10	1.61 ± 0.05	28.01 ± 1.89	48.86 ± 6.11	49.88 ± 2.89	65.82 ± 3.58	26.98	82.91
SAR	1.24 ± 0.11	21.92 ± 1.36	26.16 ± 3.85	53.95 ± 1.33	115.71 ± 8.72	105.33 ± 4.81	46.97	86.90
Salt content (mg kg^−1^)	569.67 ± 16.55	987.64 ± 20.03	10,328.51 ± 174.35	6367.49 ± 110.75	14,311.78 ± 143.93	8902.97 ± 109.56	5403.94	78.19
Salinity/sodicity class	Normal soil	Sodic soil	Sodic soil	Sodic soil	Sodic soil	Sodic soil	–	–
Soil code number	NS	SS1	SS2	SS3	SS4	SS5	–	–

ECe, ESP, SAR is electrical conductivity of saturated paste extract, exchangeable sodium percent, sodium adsorption ratio of saturated paste extract, respectively; SD is standard deviation, CV is coefficient of variation; CL, AC, PA, CH, SG, BL are different plant communities and stand for cropland, *Aneurolepidium Chinese*, *Phragmites australics*, *Chloris virgata*, *Suaeda glauca,* and bare land of saline-sodic soil, respectively; NS means normal soil, SS1–SS5 stand for different degree of sodic soil. The value of soil property parameters expressed by average ± standard deviation (n = 3).

The rest of the soil samples were used for determining their chemical properties. Soil pH was found by a pH meter (model: FiveEasy Plus in Mettler Toledo) for a slurry consisting of 1:5 (*w*/*v*) soil/distilled water. Soil electrical conductivity (EC_e_) was determined by measuring the saturated paste extract^42^. All soluble salt ions were measured in a 1:5 soil–water suspension. The total soluble carbonate (CO_3_^2−^) in soil, expressed as the CaCO_3_-equivalent (CCE), was found by a rapid-titration method, and the bicarbonate (HCO_3_^−^) content were measured by the neutral titration method^42^. The cation exchange capacity (CEC) was measured using sodium acetate (1 M NaOAc) at pH 8.2^43^. Exchangeable Na^+^ (ENa^+^) was determined by the CaCO_3_–CO_2_ exchange neutral titration method^44^. Soluble sodium ions (Na^+^) and potassium ions (K^+^) were measured using flame photometry^45^, and the soluble calcium (Ca^2+^) and magnesium (Mg^2+^) were measured in the same extract by atomic absorption spectrometry^42^. Chloridion (Cl^−^) content was determined by titration of sliver nitrate, and sulfion (SO_4_^2−^) content was found by the barium sulfate turbid metric method^46^. The total salt content (SC) was calculated as the sum of the measured cations and anions. Exchangeable sodium percentage (ESP) and sodium adsorption ratio (SAR) were calculated using measured soluble cations^47^:

1 $$ ESP = \frac{{ENa^{ + } }}{CEC} \times 100\% $$

2 $$ SAR = \frac{{Na^{ + } }}{{\sqrt {\frac{1}{2}\left( {Ca^{2 + } + Mg^{2 + } } \right)} }} $$

where *ENa*^+^ is the content of exchangeable sodium (mg kg^−1^), *CEC* is the cation exchangeable capacity (mg kg^−1^), and *Na*^+^, *Ca*^2+^ and *Mg*^2+^ are the concentrations (mg kg^−1^) of these ions, respectively.

The selected soils were classified by the US Salinity Laboratory Staff^48^ numerical criteria for salt-affected soils^48^. Based on this scheme, a soil having a EC ≥ 4 dS m^−1^ and SAR > 13 is categorized as a saline-sodic soil, those having a EC ≥ 4 dS m^−1^ and SAR < 13 are defined as saline soils, and those with a EC < 4 dS m^−1^ and SAR ≥ 13 are classed as sodic (or alkaline) soils.

### Calculation of parameters of PSD

In this study, we used a volume-based model to calculate the fractal dimension (*D*) of PSD^9^.

3a $$ \frac{{V_{{{\text{r}} < {\text{R}}_{{\text{i}}} }} }}{{V_{{\text{T}}} }} = \left( {\frac{{R_{{\text{i}}} }}{{R_{\max } }}} \right)^{3 - D} $$

where r is the soil particle size (μm), *R*_i_ is the soil particle of some grade (μm), *V*_r<Ri_ is the cumulative volume of particle sizes less than *R*_i_ (%), *V*_T_ is the sum volume of soil particles (%), *R*_max_ is the maximum soil particle size (μm), and *D* is the fractal dimension.

Taking the logarithm of both sides in Eq. (3a), we derived Eq. (3b) below to solve for *D*. Scatter diagrams are produced, with the left side of Eq. (3b) being the *y*-axis and the right side the *x*-axis. We then used least-squares regression for linear fitting, where 3-*D* was equal to the slope of the fitted straight line. We can derive the *D* value from the Eq. (3c).

3b $$ \lg \left( {\frac{{V_{{{\text{r}} < {\text{R}}_{{\text{i}}} }} }}{{V_{{\text{T}}} }}} \right) = \left( {3 - D} \right)\lg \left( {\frac{{R_{{\text{i}}} }}{{R_{\max } }}} \right) $$

3c $$ D = 3 - \frac{{\lg \left( {\frac{{V_{{{\text{r}} < {\text{R}}_{{\text{i}}} }} }}{{V_{{\text{T}}} }}} \right)}}{{\lg \left( {\frac{{R_{{\text{i}}} }}{{R_{\max }^{{}} }}} \right)}} $$

The fractal dimension therefore is a function of the PSD, and the soil particle grade will reflect the range of particle sizes and evaluate the soil grade is missing or continuous. The uniformity coefficient (*Cu*) and curvature coefficient (*Cc*) were then used for determining how well or poorly graded were the studied soils. These parameters are calculated according to the Unified Soil Classification System (USCS) described in ASTM D2487-11 (2011)^49^:

4 $$ Cu = \frac{{d_{60} }}{{d_{10} }} $$

5 $$ Cc = \frac{{d_{30}^{2} }}{{d_{60} \times d_{10} }} $$

where *d*_10_, *d*_30_ and *d*_60_ are effective, median and constrained particle size (μm), respectively.

*Cu* describes the uniformity degree of soil particle-size composition. A soil having a *Cu* ≤ 5 is called a homogenous soil, meaning the composition of the soil particle size is singular and poorly graded. In contrast, a soil is inhomogeneous when *Cu* > 5^50^. The higher the *Cu* value, the wider is the PSD. However, *Cu* exceeding a certain threshold means the possibility of a particle size of soil being missing, which is a discontinuous grade and must be judged in conjunction with *Cc*. Experience shows that when *Cu* > 5 and 1 < *Cc* < 3, then the soil is well graded; otherwise, it is poorly graded^50^.

### Statistical analysis

The statistical analyses were performed using the SPSS 19.0 statistic software package (SPSS, Inc., Chicago, IL, USA). A correlation analysis was applied to determine the correlation between fractal dimension and soil properties. Significant differences were evaluated at the 0.05 and 0.01 levels (two-tailed: significant at *P* < 0.05, very significant at *P* < 0.01). The figures were created using the Origin Pro 9.0 software (Origin Lab Inc., Northampton, MA, USA).

## Results

### Soil properties

**Figure 2:**
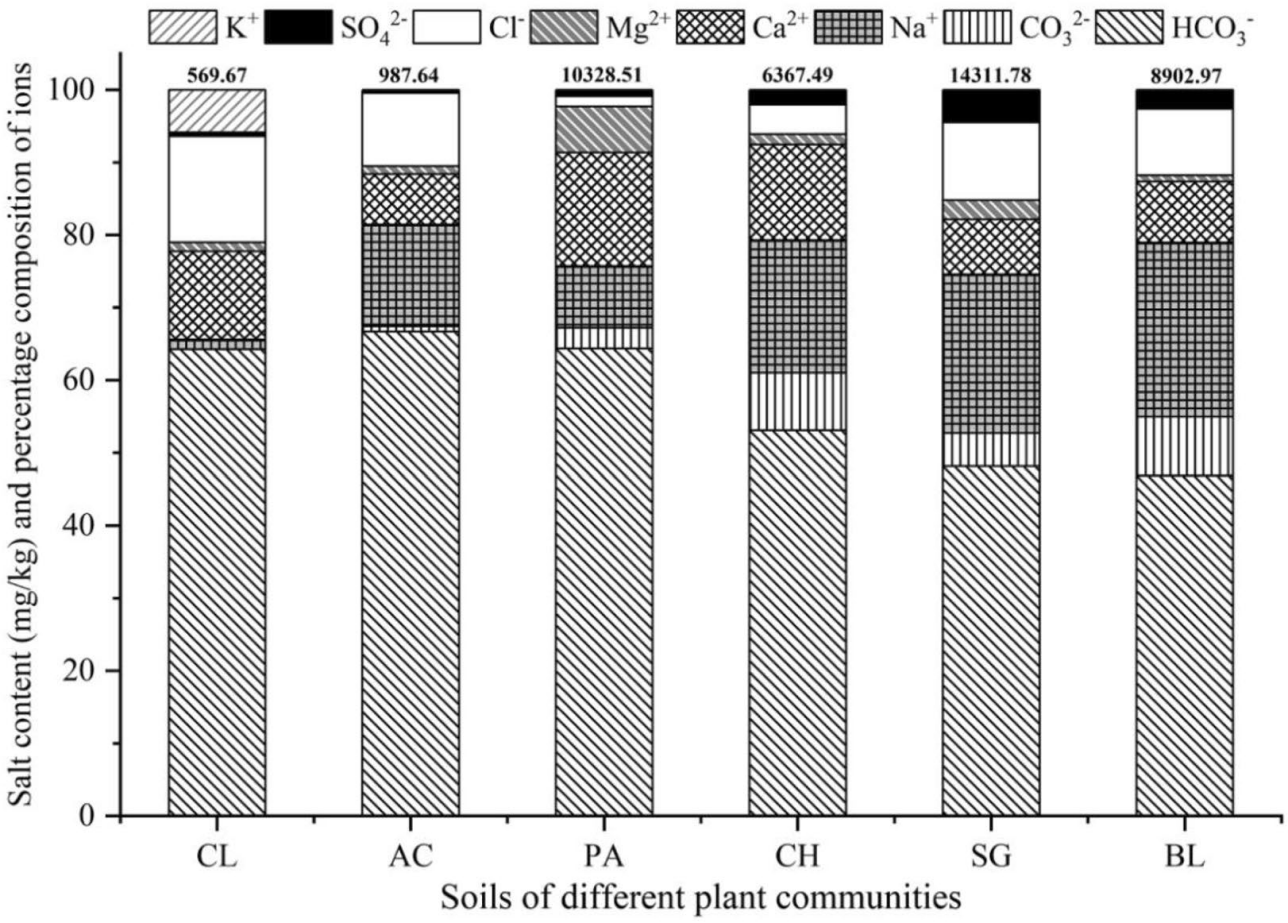
Salt content and percentage composition of salt ions in different plant communities. CL, AC, PA, CH, SG, BL are different plant communities and stand for cropland, *Aneurolepidium Chinese*, *Phragmites australics*, *Chloris virgata*, *Suaeda glauca*, and bare land of saline-sodic soil, respectively; Na^+^, K^+^,Ca^2+^, Mg^2+^, Cl^−^, SO_4_^2−^, CO_3_^2−^, HCO_3_^−^ represent the percentage content of Na^+^, K^+^, Ca^2+^, Mg^2+^, Cl^−^, SO_4_^2−^, CO_3_^2−^, and HCO_3_^−^, respectively.

The characteristics of the distribution of anions and cations and the statistics describing the selected soils’ properties are provided in Fig. 2 and Table 1. As shown in Fig. 2, HCO_3_^−^ was the major anion in the study area, with percentages ranging from 46.87 to 66.71% and an average of 57.24%. This is followed by Na^+^, whose content ranges from 1.35 to 24.03%, with an average of 14.68%, and Ca^2+^, with a content ranging from 6.97 to 15.63% and an average of 10.65%. Cl^−^ content was slightly less than Ca^2+^, with an average of 8.33%. The average content of the other ions considered was CO_3_^2−^ (4.02%) > Mg^2+^ (2.28%) > SO_4_^2−^ (1.68%) > K^+^ (1.11%). These results therefore indicate that sodium bicarbonate is the main salt component.

Table 1 shows that the selected soils have a pH ranging from 8.68 to 10.35, indicating were alkaline. Except for the ESP value for BL, which was greater than that for SG, the ECe, ENa^+^, SAR and ESP values for the six different sodic soils all display the same trend, where for each parameter value, CL < AC < PA < CH < BL < SG. In view of salt content, there were relatively low values for CL and AC (569.67 and 987.64 mg/kg, respectively), while the other four soils showed values ranging from 6367.49 to 14,311.78 mg/kg. The coefficient of variation (*CV*) represents the heterogeneity of the distribution of the soil properties. We note that the *CV* of CL is the lowest (6.85%), a product of the low-variability between its soil properties, while *CV* for the other soil types ranged from 78.19 to 89.01%, hence representing a moderate level of variability.

The studied soils were therefore classified, following the scheme of the US Salinity Laboratory Staff^48^, as being normal soils (cropland, coded as NS), and sodic soils (the soils of *Aneurolepidium chinese*, *Phragmites australics*, *Chloris virgata*, and the bare land of saline-sodic soil, coded as SS1, SS2, SS3, SS4, and SS5, respectively).

#### Fractal dimension of the particle-size distribution

The PSD of six soils are shown in Table 2. The PSD for the different soils are similar, with fine sand being the most common particle, followed by silt, coarse sand and clay. The predominant soil particles were fine sand and silt, with percentages ranging from 49.48 to 77.78% and 10.66 to 34.23%, respectively. The coarse sand and clay content were relatively low, ranging from 4.97 to 15.45% and 2.4 to 10.68%, respectively. For clay, silt and sand, the soils’ ranks (following the classification outlined in the previous section) were SS4 > SS2 > SS5 > SS1 > SS3 > NS, SS3 > NS > SS1 > SS5 > SS2 > SS4, and NS > SS3 > SS1 > SS5 > SS4, respectively. These results showed that the sand content was very high and that the clay and silt contents were relatively low. According to the classification system of the United States Department of Agriculture, the selected soils correspond to four textural classes: NS was a typical fine sand, SS1 and SS3 were loamy fine sand, SS2 and SS5 were fine sandy loam, and SS4 was very fine sandy loam.

**Table 2:** Soil particle-size distribution in different plant communities.

Code	Soil particle size distribution/%				Soil textural class	*Cu*	*Cc*
	Coarse sand/mm	Fine sand/mm	Silt/mm	Clay/mm			
	2–0.25	0.25–0.05	0.05–0.002	< 0.002			
NS	15.45 ± 0.47	71.49 ± 0.54	10.66 ± 0.15	2.40 ± 0.13	Fine sand	5.87	2.11
SS1	7.85 ± 0.25	68.65 ± 0.49	18.92 ± 0.22	4.58 ± 0.21	Loamy fine sand	16.64	4.99
SS2	8.96 ± 0.37	58.99 ± 1.03	24.09 ± 1.17	7.96 ± 0.82	Fine sandy loam	40.32	6.69
SS3	4.97 ± 0.14	77.78 ± 0.85	13.52 ± 0.35	3.73 ± 0.11	Loamy fine sand	9.09	3.35
SS4	5.61 ± .0.14	49.48 ± 0.52	34.23 ± 0.60	10.68 ± 0.32	Very fine sandy loam	40.67	2.99
SS5	7.79 ± 0.19	64.98 ± 0.41	21.23 ± 0.29	6.02 ± 0.10	Fine sandy loam	31.26	8.74

NS means normal soil, SS1, SS2, SS3, SS4, SS5 stand for different degree of sodic soil; *Cu* and *Cc* stand for uniformity coefficient and curvature coefficient, respectively. Soil particle percentage content expressed by average value ± standard deviation (n = 3).

**Figure 3:**
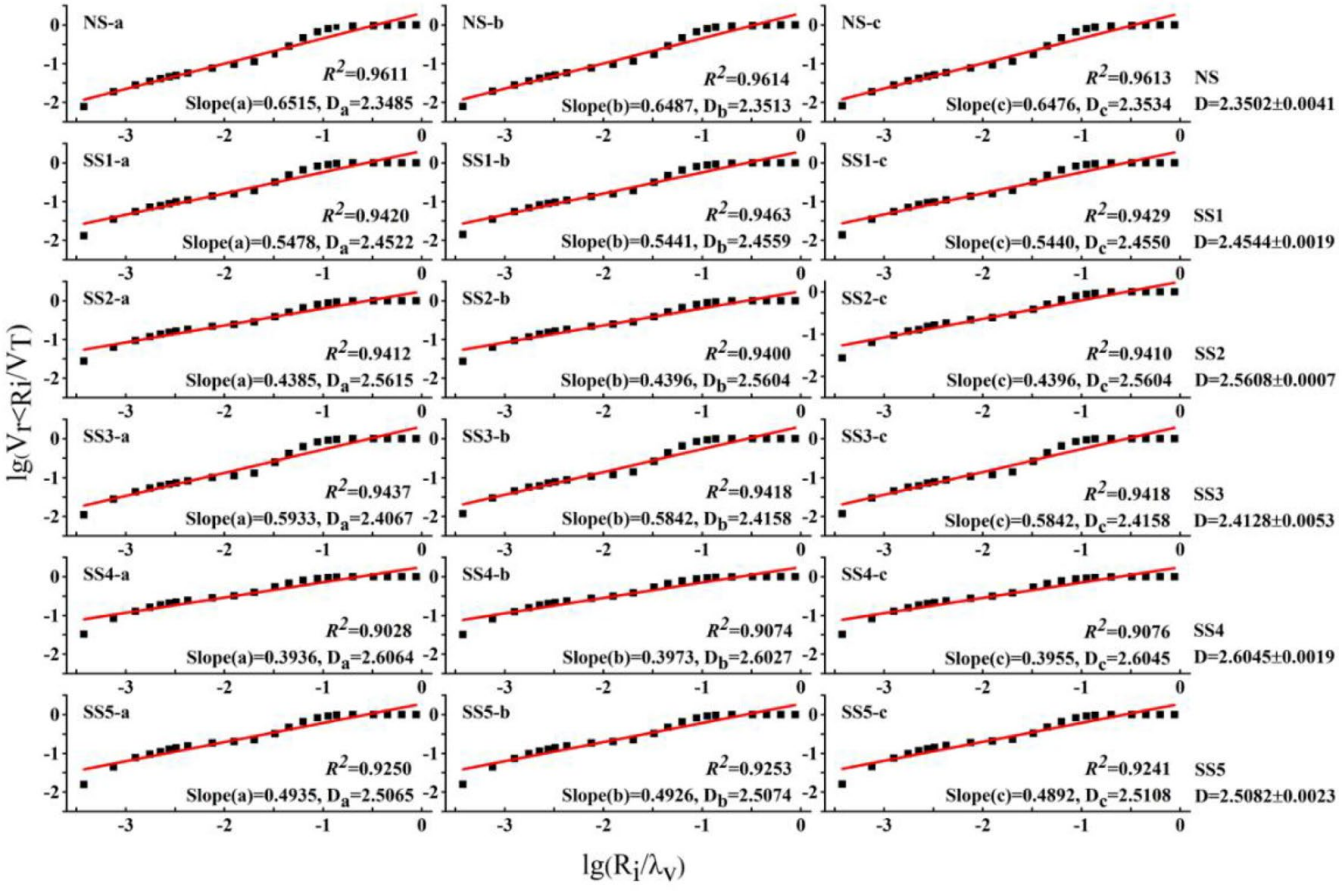
Relationship between lg(*R*_*i*_*/λ*_*v*_) and lg(*V*_*r*<*Ri*_*/V*_*T*_) for different sodic soils. NS is normal soil, SS1, SS2, SS3, SS4, SS5 stand for different degree of sodic soil; R^2^ is determination coefficient; D represents fractal dimension and equals to 3 minus the slope. “-a”, “-b”, and “-c” stand for three samples of each soil, respectively.

A linear regression analysis considering Eq. (3) was conducted, with lg(*R*_i_*/λ*_v_) as the x-axis and lg(*V*_r<Ri_*/V*_T_) as the y-axis, from which the fractal dimension of the PSD was calculated. As shown in Fig. 3, there was a strong correlation between lg(*R*_i_*/λ*_v_) and lg(*V*_r<Ri_*/V*_T_), with correlation coefficients (*r*) greater than 0.9. Results for estimating the fractal dimension ranged from 2.35 to 2.60, with an average of 2.48. The lowest value of *D* (2.35) corresponded to the soil type NS, which had the highest sand content (86.94%), but the lowest clay (2.40%) and silt (10.66%). The highest D (2.60) was found in the soil SS4, which had the highest content of clay (10.68%) and silt (34.23%) of the studied soils.

**Figure 4:**
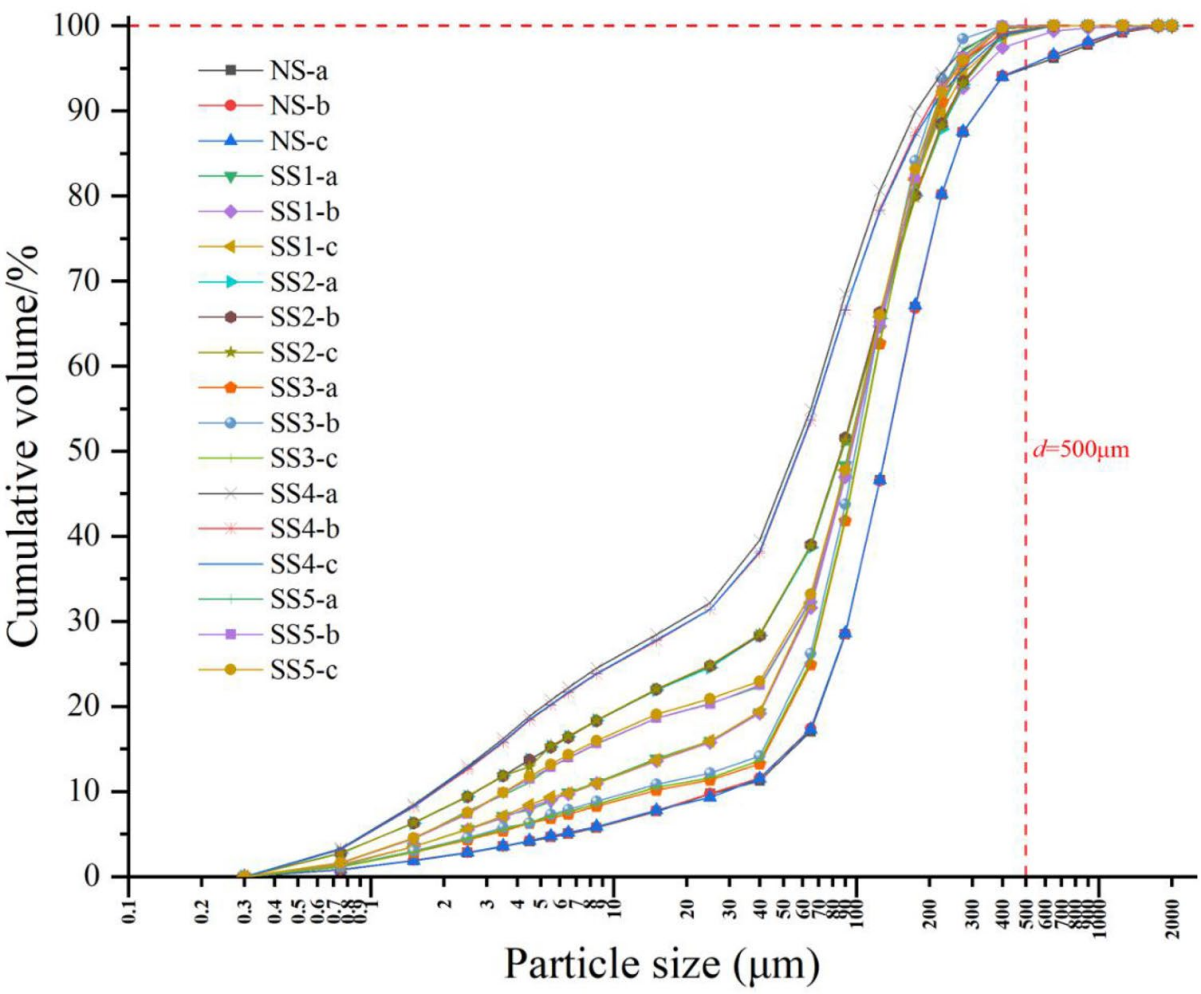
Relationship between soil particle diameter and cumulative volume. NS is normal soil, SS1, SS2, SS3, SS4, and SS5 stand for different degree of sodic soil, respectively; “-a”, “-b”, and “-c” stand for three samples of each soil, respectively.

For further analysis of the particle-size distribution, the *Cu* and *Cc* parameters (Eqs. 4 and 5) were used to determine whether the soil particle-size distribution was continuous. As shown in Table 2, *Cu* was between 5.87 and 40.67, hence greater than 5. *Cc* ranged from 2.11 to 8.74, with the *Cc* values for NS and SS4 being less than 3. Overall, NS and SS4 satisfy the condition *Cu* > 5 and 1 < *Cc* < 3. Hence, the soils NS and SS4 are considered to be well graded, and the other soils poorly graded, based on their *Cc* values. However, if the *Cu* value is much greater than 5, it indicates that this soil is likely to be missing part of the particle-size distribution. This result can also be observed from the cumulative distribution curve of soil particle size, as shown in Fig. 4. We note that the NS curve gradually changed within the range of 0–2000 μm, while the other curves tended to be almost horizontal by the point when *d* was approximately equal to 500 μm. This means that the component of the soil with a size d > 500 μm was fairly small, and effectively missing in all soils, except for NS.

#### Relationship between particle-size distribution and fractal dimension

**Figure 5:**
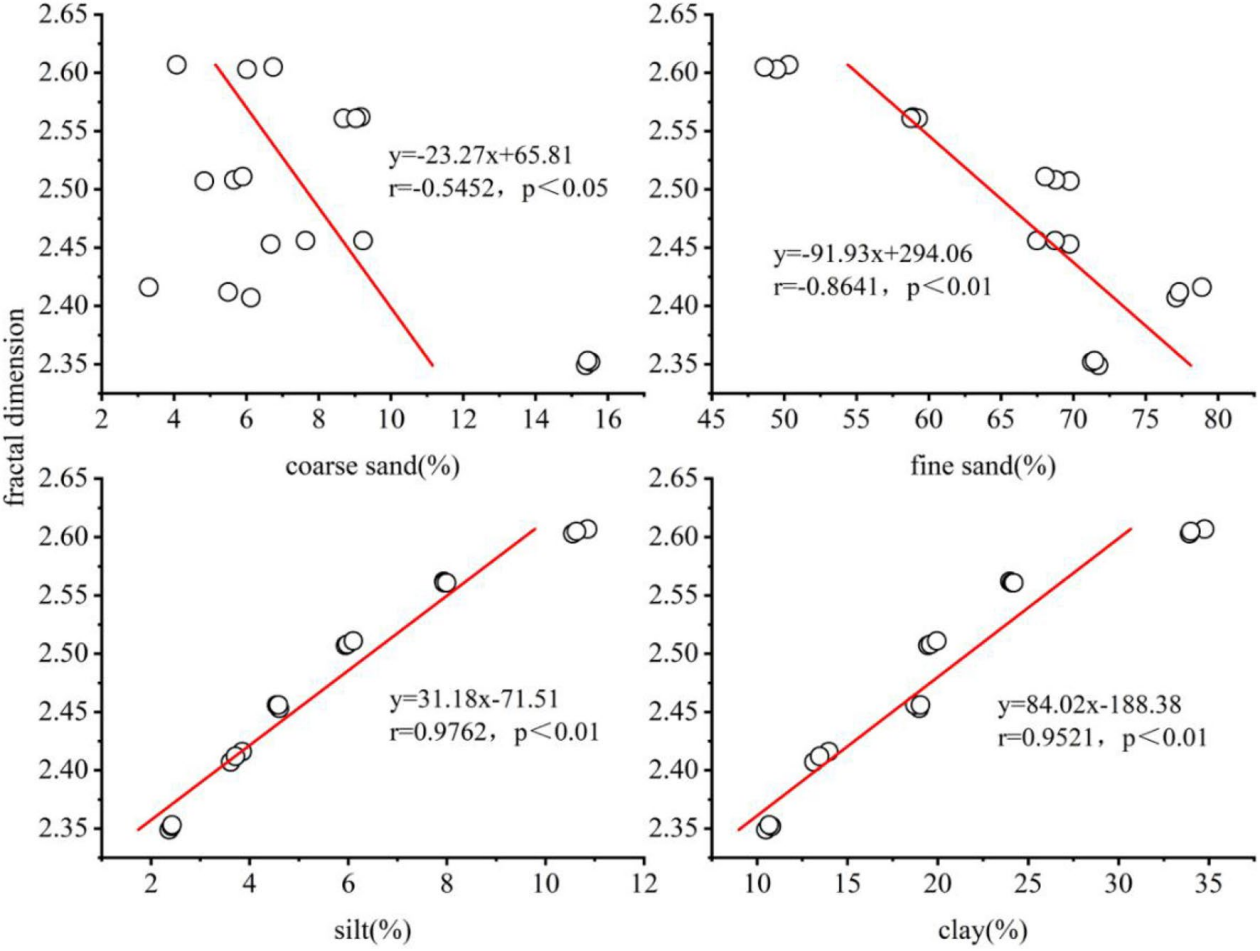
Relationship between fractal dimension and contents of soil particles. Coarse sand, fine sand, silt and clay is the coarse sand, fine sand, silt and clay content (%), respectively. Equation derived from the liner regression fitting; *r* is the correlation coefficient.

A simple linear regression analysis was performed to establish the relationships between the inferred *D* of the PSD, and with sand, silt and clay content. As shown in Fig. 5, the *D* of the PSD showed a significant negative correlation with coarse sand (*r* =  − 0.5452, *P* < 0.05), a very significant negative correlation with the fine sand content (*r* =  − 0.8641, *P* < 0.01), and a very significant positive correlation with the silt and clay content (*r* = 0.9726, *P* < 0.01 and *r* = 0.9526, *P* < 0.01, respectively).

#### Correlation between fractal dimension of soil and its properties

The correlation coefficients and significance between the *D* of the PSD and properties of the soils are listed in Table 3. The correlation analysis indicated that *D* has a significant positive correlation with the total salt content (SC) (*P* < 0.05) and a very significant positive correlation with HCO_3_^−^ content (*P* < 0.01). Similarly, the SC and HCO_3_^−^ contents displayed a significant negative correlation with sand content (*P* < 0.05), and a significant positive correlation with the silt (*P* < 0.05) and clay contents (*P* < 0.01). However, for all studied soil samples, there was no clear correlation between *D* and the other chemical parameters considered, such as Na^+^, ENa^+^, ESP and CEC.

**Table 3:** Correlation between soil fractal dimension (*D*) and soil properties.

Item	D	Na^+^/mg kg^−1^	ENa^+^/cmol kg^−1^	Ca^2+^/mg kg^−1^	CO_3_^2−^/mg kg^−1^	HCO_3_^−^/mg kg^−1^	SC/mg kg^−1^	CEC/cmol kg^−1^	ESP/%	SAR	Sand/%	Silt/%	Clay/%
D	1	0.663	0.697	0.673	0.419	0.885**	0.872*	0.749	0.490	0.372	− 0.961**	0.952**	0.974**
Na^+^/mg kg^−1^		1	0.937**	0.236	0.899**	0.669	0.858*	0.343	0.866*	0.861*	− 0.669	0.666	0.667
ENa^+^/cmol kg^−1^			1	0.428	0.794*	0.750	0.890**	0.343	0.926**	0.681	− 0.663	0.650	0.691
Ca^2+^/mg kg^−1^				1	0.074	0.867*	0.693	0.251	0.344	− 0.122	− 0.575	0.539	0.667
CO_3_^2−^/mg kg^−1^					1	0.483	0.685	0.018	0.885**	0.963**	− 0.354	0.351	0.363
HCO_3_^−^/mg kg^−1^						1	0.956**	0.417	0.625	0.343	− 0.825*	0.799*	0.886**
SC/mg kg^−1^							1	0.424	0.771*	0.572	− 0.836*	0.817*	0.878**
CEC/cmol kg^−1^								1	0.063	0.083	− 0.815	0.834	0.854
ESP/%									1	0.743	− 0.392	0.375	0.345
SAR										1	− 0.344	0.350	0.322

D stands for fractal dimension of particle size distribution; ENa^+^, SC and CEC equal to exchangeable sodium, salt content and cation exchange capacity, respectively; Na^+^, ENa^+^, Ca^2+^, CO_3_^2−^, HCO_3_^−^, SC and CEC stand for the Na^+^, ENa^+^, Ca^2+^, CO_3_^2−^, HCO_3_^−^, SC, and CEC content, respectively; ESP and SAR is exchangeable sodium percent, sodium adsorption ratio of saturated paste extract, respectively; Sand, silt and clay is the sand, silt and clay content (%), respectively.

**Means significant at the 0.01 level, * means significant at the 0.05 level.

## Discussion

### The fractal dimension of particle-size distribution

Soil PSD is a basic fundamental physical parameter and is commonly used for classifying and judging soil texture^51^. Previous studies have shown that the fractal dimension of debris flow and glacial material is approximately 2.6, and that of river and lake sediments is generally less than 2.6^52^. The fractal dimension of soils with finer textures and abundant nutrients typically ranges from 2.60 to 2.80, and those with coarse sand and poor structure range from 1.83 to 2.64^53^. In this study, the samples were collected from a flood plain, and had a fractal dimension ranging from 2.35 to 2.61, with an average of 2.48. Hence, our results are in line with previous findings. Furthermore, Liu X et al. (2009) reported that the fractal dimension of well-structured soils is approximately 2.75^54^. However, since the sampled soils in this study had fractal dimension of less than 2.75, this suggests they have poor structure and are missing part of particle-size composition. This can be confirmed from Fig. 4, which shows that for most samples, the cumulative volume reaches approximately 98% when the soil particle diameter reaches 500 μm, with the cumulative volume of particle diameters between 500 and 2000 μm accounting for only 2%. The cumulative volume of the particle sizes ranging between 50 and 250 μm accounted for 49.48 to 77.78%, indicated fine sand with a majority, hence leading to a coarse texture and poor structure, which are not conducive to soil water and nutrients conservation.

Fractal dimension reflected a significant correlation with alkaline soil particle-size distribution. Here, fractal dimension showed a very significant negative correlation with sand content (*P* < 0.01), and a significant positive correlation with silt and clay content (*P* < 0.01). Zhu et al.^55^ investigated the influence of adding fly ash and montmorillonite-enriched sandstone on the particle-size distribution in a alkaline soil with loamy fine sandy texture, and the results indicated that the fractal theory could be used to quantify the relationships between fractal dimension and particle-size distribution in amended soil. Our results confirmed that the fractal dimension of the PSD increases as the particle size becomes finer. Several previous studies of soils not affected by salt and our study in alkaline soils have revealed the consistent correlation between fractal dimension and the particle-size distribution^7,18^. For example, the negative correlation between sand content and fractal dimension indicates that the fractal dimension would decrease as the sand content increased. Such correlations suggest that fractal dimension has the potential to be a useful tool for simulating and characterizing the PSD of the soils^7^.

### Fractal dimension and soil properties

Considering non-saline and non-alkaline soils, numerous studies have shown that there are significant statistical correlations between the fractal dimension of the PSD of soils, and their physicochemical properties^51^. Considering the analysis of the relationships between fractal dimension and alkalinity properties of soils carried out in this work, our results indicate that the fractal dimension showed a significant positive correlation with salt content (*P* < 0.05), and a significant positive correlation with the HCO_3_^−^ (*P* < 0.01). While the study area in this work was located at a transition zone between semi-arid to semi-humid environments with the salt component dominated by bicarbonate and carbonate, Zhao et al.^32^ considered an arid zone whose salt content was mainly comprised of sulfate and chloride. They also found fractal dimension and salt content were positively correlated, and in agreement with our results, determined that the fractal dimension increased with fine particle content, but decreased with increasing coarse particles content. Hu et al.^56^ considered an arid mulched drip irrigation system, and determined that the soil PSD dominated the soil water and salt distribution, especially the surface soil salt content and the deep soil water content.

However, alternate results have also been presented in the literature. For example, Gui et al.^31^, who investigated the vegetation and soil conditions from elevations of 1960 to 4070 m on the north slope of the Kunlun Mountains, China, found that the fractal dimension was negatively correlated with total salt content. As described by Gui et al.^31^, the soil fine fractions increased gradually with increasing elevation. Therefore, we inferred that soil salt is mainly concentrated in the coarse particles and this is likely to have a close relationship with the soil’s parent material. Nonetheless, overall, the fractal dimension has been seen to have a close relationship with soil salt content. However, no significant correlation was been found between fractal dimension and exchangeable Na^+^ in this study.

In this study, salt content and HCO_3_^−^ increased with the increasing fine particle content (clay and silt particles) and decreased with increasing sand contents. That is, salt content and HCO_3_^−^ content were inversely proportional to the particle size. In a chloride–sulfate and sulfate soil, Zhao et al.^32^ similarly showed that both the content of HCO_3_^−^ and SO_4_^2−^ show a positive correlation with clay and silt content, and a negative correlation with sand content at different soil depths^32^. Other studies have shown that soil nutrients and metal ions tend to be aggregated with fine particles (silt and clay) rather than with coarser material^57,58^. Hence, when fine particle content is higher, the greater is the specific surface area and thus the stronger the adsorption^59^. However, no significant correlation has been found between exchangeable Na^+^, Ca^2+^ and particle-size distribution in this study.

## Conclusions

The results of this work have found that the fractal dimension of the particle-size distribution of the studied soils have a close relationship with alkalinity properties of soils and soil texture. A significant negative correlation was observed between the fractal dimension and both coarse sand (*P* < 0.05) and fine sand content (*P* < 0.01), and a positive correlation between fractal dimension and silt (*P* < 0.01) and clay content (*P* < 0.01). In other words, there is an increase in the fractal dimension as the soil texture changes from coarse to fine. Salt content, particularly HCO_3_^−^ content, was inversely proportional to soil particle size. A correlation analysis showed that the typical characteristics of alkaline soil, such as the Na^+^, ENa^+^ and Ca^2+^ content, had no obvious relationship with fractal dimension. However, a significant positive relationship was shown between fractal dimension and salt content (*P* < 0.05), and in particular, a very significant positive correlation with HCO_3_^−^ content (*P* < 0.01). Our results therefore indicate that the fractal dimension of soil can be used as an indicator of its alkalinity and texture.

## References

[1] Mandelbort, B. B. The Fractal Geometry of Nature. Freeman M, San Francisco, pp. 45–256 (1982).

[2] Turcotte DL (1986). Fractals and fragmentation. J. Geophys. Res..

[3] Rieu M, Sposito G (1991). Fractal fragmentation, soil porosity and soil water properties: I. theory. Soil Sci. Soc. Am. J..

[4] Tyler SW, Wheatcraft SW (1992). Fractal scaling of soil particle-size distributions: analysis and limitations. Soil Sci. Soc. Am. J..

[5] Yang PL, Luo YP, Shi YC (1993). Fractal features of soil defined by grain weight distribution. Chin. Sci. Bull..

[6] Song XY, Li HY (2011). Fractal characteristics of soil particle-size distribution under different landform and land-use types. Adv. Mater. Res..

[7] Mohammadi M, Shabanpour M, Mohammadi MH, Davatgar N (2019). Characterizing spatial variability of soil textural fractions and fractal parameters derived from particle size distributions. Pedosphere.

[8] Martın MA, Montero E (2002). Laser diffraction and multifractal analysis for the characterization of dry soil volume-size distributions. Soil Tillage Res..

[9] Wang GL, Zhou SL, Zhao QG (2005). Volume fractal dimension of soil particles and its applications to land use. Acta Pedol. Sin..

[10] Zhao WJ, Cui Z, Ma H (2017). Fractal features of soil particle-size distributions and their relationships with soil properties in gravel-mulched fields. Arab. J. Geosci..

[11] Yang JL (2008). Comparison of mass and volume fractal dimension of soil particle size distribution. Acta Pedol. Sin..

[12] Chari MM, Mohammad RDG (2019). Evaluating fractal dimension of the soil particle size distributions and soil water retention curve obtained from soil texture components. Arch. Agron. Soil Sci..

[13] Keller T, Håkansson I (2010). Estimation of reference bulk density from soil particle size distribution and soil organic matter content. Geoderma.

[14] Ding YD, Zhao Y, Feng H, Si BC, Robert LH (2016). A user-friendly modified pore-solid fractal model. Sci. Rep..

[15] Zhao WJ, Cao TH, Dou PX, Sheng J, Luo MQ (2019). Effect of various concentrations of superabsorbent polymers on soil particle-size distribution and evaporation with sand mulching. Sci. Rep..

[16] Nichols PWB, Lucke T (2017). A detailed analysis of sediment particle sizes and clogging in permeable pavements. Clean-Soil Air Water.

[17] Xu GC, Li ZB, Li P (2013). Fractal features of soil particle-size distribution and total soil nitrogen distribution in a typical watershed in the source area of the middle Dan River, China. CATENA.

[18] Li K (2018). Fractal features of soil particle size distributions and their potential as an indicator of *Robinia pseudoacacia* invasion. Sci. Rep..

[19] Su ZH, Zhao HL, Wen ZZ, Zhang TH (2004). Fractal features of soil particle size distribution and the implication for indicating desertification. Geoderma.

[20] Ersahin S, Gunal H, Kutlu T, Yetgin B, Coban S (2006). Estimating specific surface area and cation exchange capacity in soils using fractal dimension of particle-size distribution. Geoderma.

[21] Qi F (2018). Soil particle size distribution characteristics of different land-use types in the Funiu mountainous region. Soil Tillage Res..

[22] Feng X, Qu JJ, Tan LH, Fan QB, Niu QH (2019). Fractal features of sandy soil particle-size distributions during the rangeland desertification process on the eastern Qinghai-Tibet Plateau. J. Soil Sediments.

[23] Yu Y, Wei W, Chen LD, Feng TJ (2016). land preparation and vegetation type jointly determine soil conditions after long-term land stabilization measures in a typical hilly catchment, Loess Plateau of China. J. Soil Sediments.

[24] Qadir M, Ghafoor A, Murtaza G (2000). Amelioration strategies for saline soils: a review. Land Degrad. Dev..

[25] Sumner ME (1993). Sodic soils—new perspective. Aust. J. Soil Res..

[26] Frenkel H, Goertzen JO, Rhoades JD (1978). Effects of clay type and content, exchangeable sodium percentage, and electrolyte concentration on clay dispersion and soil hydraulic conductivity. Soil Sci. Soc. Am. J..

[27] Ding WQ (2019). How the particle interaction forces determine soil water infiltration: specific ion effects. J. Hydrol..

[28] Sevda A, Hossein G, Cheng RC, Petra M (2016). Salt-affected soils, reclamation, carbon dynamics, and biochar: a review. J. Soil Sediments.

[29] Gangwar P, Singh R, Trivedi M, Tiwari RK, Shukla V, Kumar N (2020). Sodic soil: management and reclamation strategies. Environmental Concerns and Sustainable Development.

[30] Ding WQ (2019). The effect of interactions between particles on soil infiltrability. J. Soil Sediments.

[31] Gui DW (2010). Ordination as a tool to characterize soil particle size distribution, applied to an elevation gradient at the north slope of the Middle Kunlun Mountains. Geoderma.

[32] Zhao Y, Feng Q, Yang HD (2016). Soil salinity distribution and its relationship with soil particle size in the lower reaches of Heihe River, Northwestern China. Environ. Earth Sci..

[33] Yang F, Zhang GX, Yin XR, Liu ZJ (2011). Field-scale spatial variation of saline-sodic soil and its relation with environmental factors in Western Songnen Plain of China. Int. J. Environ. Res. Public Health.

[34] Yang F (2016). Variations on soil salinity and sodicity and its driving factors analysis under microtopography in different hydrological conditions. Water.

[35] Yu JB (2010). Biogeochemical characterizations and reclamation strategies of saline sodic soil in northeastern China. Clean-Soil Air Water.

[36] Smith ME, Facelli JM, Cavagnaro TR (2018). Interactions between soil properties, soil microbes and plants in remnant-grassland and old-field areas: a reciprocal transplant approach. Plant Soil.

[37] Niu YJ, Yang SW, Zhou JW (2019). Vegetation distribution along mountain environmental gradient predicts shifts in plant community response to climate change in alpine meadow on the Tibetan Plateau. Sci. Total Environ..

[38] Füzy A, Tóth T, Biró B (2008). Soil-plant factors, others than the type of salt-specific anions are affecting the mycorrhiza colonisation of some halophytes. Community Ecol..

[39] He Q, Silliman BR, Cui B (2017). Incorporating thresholds into understanding salinity tolerance: a study using salt-tolerant plants in salt marshes. Ecol. Evol..

[40] Gao Q, Li JD, Zheng HY (1996). A dynamic landscape simulation model for alkaline grasslands on Songnen Plain in northeast China. Landsc. Ecol..

[41] Xin XP, Gao Q, Li ZQ, Yang ZY (1996). Partitioning the spatial and environmental variations of plant community structure of alkaline grassland on Songnen Plain. Acta Bot. Sin..

[42] Rayment GE, Higginson FR (1992). Australian Laboratory Handbook of Soil and Water Chemical Methods.

[43] Chapman, H. D. Cation exchange capacity. In: Black C A (Ed.), Methods of Soil Analysis Part 2, Agronomy Am. Inst, Madison, WI, 891–901 (1965).

[44] Sparks, D. L. *Methods of Soil Analysis, SSSA, ASA*. Madison, Wisconsin, USA, pp.1215–1218 (1996).

[45] Jackson, M. L. *Soil Chemistry of Analysis*. Englewood Cliffe, New Jersy: Prentice Hall, 78 (1962).

[46] Eaton AD, Clesceri LS, Rice EW, Greenberg AF (2005). Standard Methods for the Examination of Water and Wastewater.

[47] Stewart B, Lal R (1992). Advances in Soil Science.

[48] US Salinity Laboratory Staff. Diagnosis and Improvement of Saline and Alkali Soils. USDA Handbook 60. US Government Printing Office: Washington, DC (1954).

[49] American Society for Testing and Materials (ASTM). *Standard Practice for Classification of Soils for Engineering Purposes (United Soil Classification System)*. D2487-11, ASTM, West Conshohocken, PA, USA (2011).

[50] Li XP, Liu JL, Zhang JB, Wang WP, Xin WW (2014). Analysis of fractal magnitude of soil particles in loamy Chao soils in North China Plain. Trans. CSAE.

[51] Hillel D (1980). Fundamentals of Soil Physics.

[52] Bai CG, Mu GJ, Wang J (2002). Grain-size dimensions characteristics of lacustrine sediments of Aiby Lake and the environmental significance. Arid Land Geogr..

[53] Filgueira RR, Fournier LL, Cerisola CI, Gelati P, Garcia GM (2006). Particle-size distribution in soils: a critical study of the fractal model validation. Geoderma.

[54] Liu X, Zhang GC, Heathman GC, Wang YQ, Huang CH (2009). Fractal features of soil particle-size distribution as affected by plant communities in the forested region of Moutain Yimeng, China. Geoderma.

[55] Zhu SL, Zhen Q, Zhang XC (2019). Multifractal characteristics of the pore structures of physically amended sandy soil and the relationship between soil properties and multifractal parameters. Arch. Agron. Soil Sci..

[56] Hu HC, Tian FQ, Hu HP (2011). Soil particle size distribution and its relationship with soil water and salt under mulched drip irrigation in Xinjiang of China. Sci. China Technol. Sci..

[57] Lobe I, Amenlung W, Du Preeez CC (2001). Losses of carbon and nitrogen with prolonged arable cropping from sandy soils of the South African Highveld. Eur. J. Soil Sci..

[58] Ljung K, Sekinus O, Otabbong E, Berglund M (2006). Metal and arsenic distribution in soil particle sizes relevant to soil ingestion by children. Appl. Geochem..

[59] Acosta JA, Cano AF, Arocena JM, Debela F, Martinez-Martines S (2009). Distribution of metals in soil particle size fractions and its implication to risk assessment of playgrounds in Murcia City(Spain). Geoderma.

